# Assessing cognitive impairment in an ethnically diverse cohort of oldest-old: the life after 90 study

**DOI:** 10.1007/s40520-023-02368-0

**Published:** 2023-03-04

**Authors:** Diana Petrosyan, Maria M. Corrada, Claudia H. Kawas, Asmeret Demoz, Czarina Ganzon, Oksana Popovich, Reham Gaied, Dan Mungas, Paola Gilsanz, Katharine E. Vieira, Rachel A. Whitmer, Charles DeCarli

**Affiliations:** 1 School of Medicine, Department of Neurology, University of California at Davis, 4860 Y Street, Suite 3700, Sacramento, CA 95817 USA; 2 School of Medicine, Department of Neurology, University of California at Irvine, Irvine, CA 92617 USA; 3 Kaiser Permanente Division of Research, Oakland, CA 94612 USA; 4 School of Medicine, Department of Public Health Sciences, University of California at Davis, Davis, CA 95616 USA; 5 School of Medicine, Department of Epidemiology, University of California at Irvine, Irvine, CA 92617 USA; 6 School of Biological Sciences, Department of Neurobiology and Behavior, University of California at Irvine, Irvine, CA 92617 USA

**Keywords:** Cognitive impairment, Diverse population, Oldest-old, 3MS, Epidemiology, Cognitive testing

## Abstract

**Background:**

Though dementia rates vary by racial or ethnic groups, it is unknown if these disparities remain among those aged 90 or older.

**Aims:**

To test this hypothesis, we used baseline clinical evaluation of 541 ethnically and racially diverse individuals participating in the *LifeAfter90* Study to assess how associations between core demographic characteristics and measures of physical and cognitive performance differ across the racial/ethnic groups.

**Methods:**

Participants in this study were long-term non-demented members of Kaiser Permanente Northern California. They were clinically evaluated and diagnosed with normal or impaired cognition (mild cognitive impairment and dementia) through an in-person comprehensive clinical assessment consisting of a detailed medical history, physical and neurological examination, functional, and cognitive tests.

**Results:**

The average age at enrollment was 93.0 ± 2.6 years, 62.4% female and 34.2% non-Hispanic White. At initial evaluation 301 participants had normal cognition and 165 had mild cognitive impairment (MCI) and despite screening, 69 participants were determined to have dementia. Age, education, 3MS, FAQ and CDR scores were significantly associated with cognitive impairment (normal versus MCI and dementia), but not gender. There was a significant univariate association between race/ethnicity and cognitive impairment (*p* < 0.02) being highest among Black (57.4%) and lowest among Asian (32.7%) individuals. After adjustment for age, gender, and education, however, prevalence of cognitive impairment was not influenced by race or ethnicity.

**Conclusion:**

Our results confirm the ability to reliably assess clinical diagnosis in a diverse sample of very old individuals.

**Supplementary Information:**

The online version contains supplementary material available at 10.1007/s40520-023-02368-0.

## Introduction

In the US, people aged 90 and over are the fastest growing part of the population [1]. In 2010, this group represented 4.7% of the older population (≥ 65 years) and is estimated to reach 10% by 2050, representing 2% of total US population [2].

The incidence of dementia in this age group remains controversial as studies of this age group are often limited to small study size, presence of comorbidities, and difficulties distinguishing between cognitive and non-cognitive contributors to loss of functional abilities making diagnosis of cognitive impairment challenging [3]. Despite significant variability in studies, the literature suggests that both the incidence [4] and prevalence [5] of dementing disorders continue to increase with age and are particularly high among people beyond 90 years of age. Yet, current understanding of cognitive impairment in this age group is limited to studies of predominantly non-Hispanic White (White) individuals [3, 5–7]. Studies of younger diverse populations suggest marked differences in rates of dementia by racial/ethnic groups, with higher rates among Black and lower rates among Asian individuals [8], including those over age 90 [9]. A significant gap, therefore, exists in the epidemiology of dementia particularly among underrepresented ethno-racial groups of oldest old. Understanding dementia and its risk factors among ethno-racially diverse populations is particularly important given that the proportion of non-White elderly individuals continues to increase and estimated to be about 30% of oldest-old population in the U.S. by 2050 [2].

The *LifeAfter90* Study is an ongoing prospective cohort study aimed to investigate life-course determinants of dementia incidence, cognitive decline, neuropathologic changes, and brain imaging markers in an ethnically and racially diverse cohort of individuals aged 90 years and older.

This manuscript describes the baseline clinical diagnostic evaluation of the first 541 participants enrolled into the *LifeAfter90* Study.

## Methods

### Study participants

**Fig. 1:**
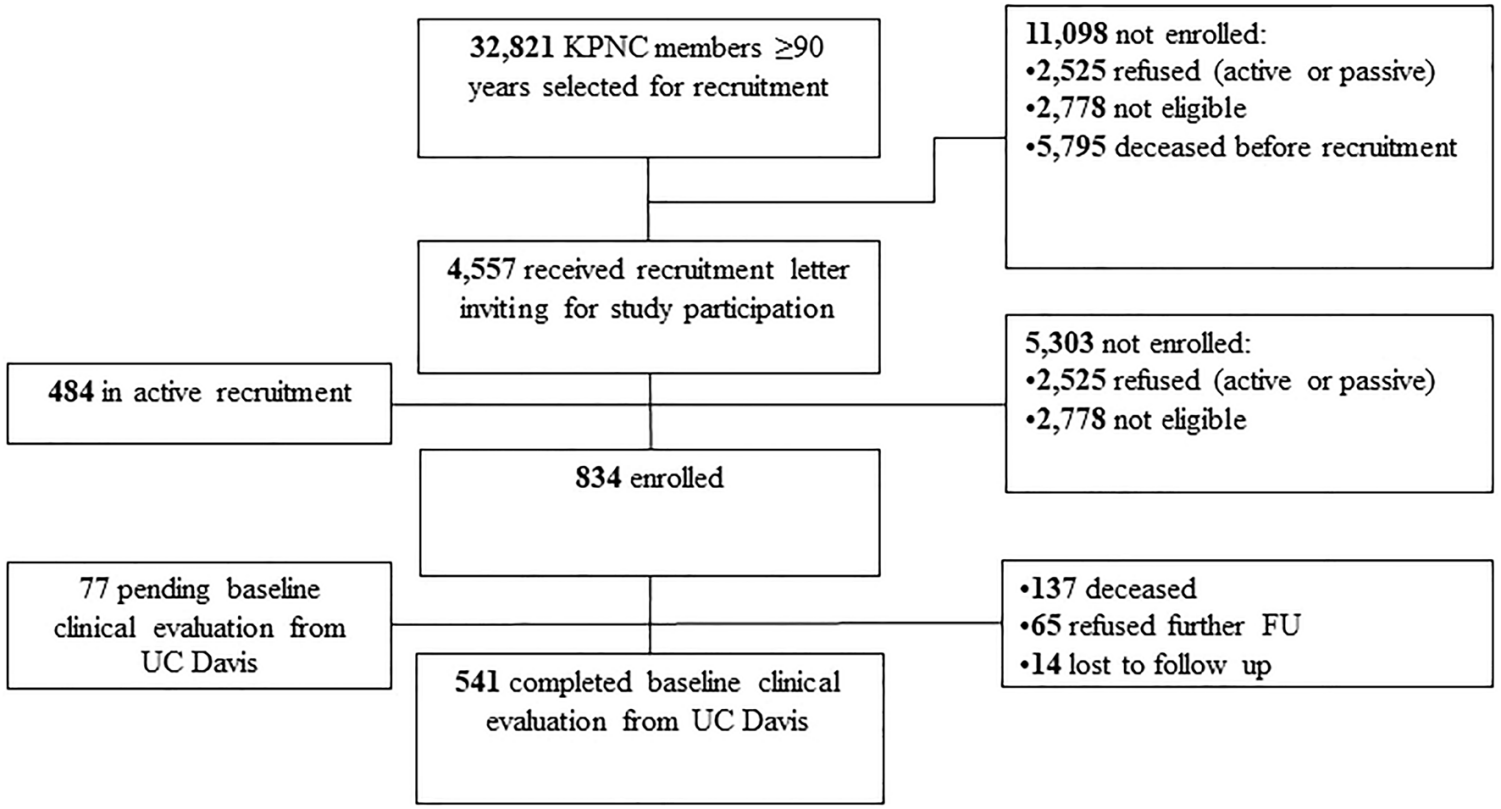
Enrollment Flow Chart

Participants in the *LifeAfter90* Study are long-term members of Kaiser Permanente Northern California (KPNC) who were 90 years or older residing in the San Francisco Bay and Sacramento areas of California. Eligible individuals were KPNC members at some point between 1964 and 1992 and spoke English or Spanish. Exclusion criteria included inability to provide informed consent or a diagnosis of dementia or other neurodegenerative disease, hospice care, or dialysis in their electronic medical record at the time of enrollment. Study enrollment began in July 2018 and is ongoing. Figure 1 presents the flow chart of the participants’ enrollment as of March 2021. This analysis includes the first 541 individuals who were enrolled and completed the baseline clinical component with UC Davis from July 2018-February 2020.

The *LifeAfter90* Study was approved by the KPNC, and UC Davis Institutional Review Boards and all enrolled participants provided informed consent.

### Clinical evaluation components

UC Davis was responsible for the clinical component of this study which included detailed clinical evaluation of all participants approximately twice yearly. Six physicians trained by a behavioral neurologist (CD) with extensive experience in diagnosing cognitive impairment and dementia, conducted the clinical examination of the study participants. To facilitate participation of individuals with limited mobility, sensory impairment, or frailty, all evaluations are done in the participants’ homes. The evaluation consisted of collecting detailed medical history including history of cognitive complaints, a physical and neurological examination, cognitive function tests (Modified Mini-Mental State (3MS) examination [10] and Clinical Dementia Rating (CDR) [11]), assessment of physical performance (Short Physical Performance Battery(SPPB) [12]) and assessment of functional impairment (Functional Activities Questionnaire(FAQ) [13]), tools commonly used in the clinical assessment of presence and severity of cognitive impairment [14, 15] (Supplementary Table S1).

### Clinical evaluation instruments

#### Modified mini-mental state exam (3MS)

The 3MS, a modified version of Mini-Mental State Exam, includes the following domains: verbal fluency, reasoning/judgment, expressive language, visual construction, immediate and delayed free verbal recall, and cued verbal recall [10]. Several studies documented its superiority to MMSE in discriminating different levels of cognitive impairment [16–19].

#### Clinical dementia rating (CDR)

The CDR was administered to the participant and the informant, if one was available at the time of the clinical evaluation. It collected information through semi-structured interviews to assess the presence and level of cognitive impairment in six domains: memory, orientation, judgment and problem solving, community affairs, home and hobbies, and personal care [11].

#### Functional activities questionnaire (FAQ)

The FAQ is a 12-item scale that collected information on instrumental activities of daily living [13]. A modified version of FAQ was used in this study enabling distinction between different causes (cognitive vs other (sensory or physical)) for functional impairment. It was administered primarily to the participant and was supplemented with the information provided by the informant, when available. The modified FAQ allowed for the assignment of separate sub-scores indicating if functional impairment was due to cognitive or non-cognitive limitations including hearing or visual impairment, changes in upper or lower extremities, or balance problems (Supplementary table S2).

#### Short physical performance battery (SPPB)

SPPB was used to assess balance, gait, and lower extremity strength in older individuals [12] known to be associated with cognitive decline [20]. The SPPB consists of 5 tests of physical ability: (1) side-by-side stand, (2) semi-tandem stand, (3) tandem stand, (4) timed walk (3 or 4 m), and 5) time to arise from a chair 5 times. Each physical activity is timed and scored by ability and time to complete, with a range of 0 indicating no ability to 12 indicating full ability at all tasks.

### Clinical diagnosis and adjudication

Diagnosis of dementia was made using the criteria of Diagnostic and Statistical Manual of Mental Disorders Fourth Edition and required (1) impairment in memory and one additional cognitive domain; (2) decline from a previous level of functioning due to cognition; and (3) presence of cognitive deficits not only during delirium [21] as updated by National Institute on Aging, Alzheimer’s Association [15]. Diagnosis of mild cognitive impairment (MCI) was consistent with Petersen’s criteria [22] and required (1) subjective complaints; (2) impairment in one or more cognitive domains; (3) no functional impairment due to cognition; and (4) no dementia. Final clinical diagnosis of the participants was determined in the following sequence: (1) at the end of each clinical evaluation, the physicians documented their initial impression based on the overall information collected during the assessment that included history, physical and neurological examination along with “bedside” cognitive and functional testing as indicated by recent recommendations [15], and assigned the participant a diagnosis of either normal cognition (NC), mild cognitive impairment (MCI), or dementia; (2) a behavioral neurologist reviewed the documented results of the clinical evaluation and adjudicated the final clinical diagnosis. Discrepancies between the initial clinical impression and final clinical diagnosis were reviewed and discussed during quality control and reliability sessions conducted monthly until the final consensus diagnosis was reached.

### Statistical analysis

The primary dependent variable was the clinical diagnosis of NC, MCI, or dementia. Due to the relatively low number of individuals with dementia in this subsample, however, clinical diagnosis was operationalized to normal or impaired cognition (MCI or dementia) in the final analysis. Independent variables of interest included demographic variables (race/ethnicity, gender, age, and education), self-described measures of general health (health perception, smoking status, medical history including presence of cardiovascular, cerebrovascular, or vascular diseases), physical (SPPB and FAQ non-cognitive scores) and cognitive performances (3MS, CDR, CDR sum of boxes (CDR SOB [23]) and FAQ cognitive scores). Race/ethnicity was captured in five categories: Asian, Black, Latino, White, and multiracial. Education was coded as three categories: achievement up to High School graduation or GED, any college education, and any graduate education. Missing values for all variables were checked for randomness using Little’s missing at random test [24] and were replaced with the mean of the variable if more than 1% of data was missing randomly for quantitative variables. Only the SPPB total score had more than 1% missing values (1.8%) and were replaced with the mean SPPB score of 6.29.

We first generated basic descriptive statistics for all participants. Differences between the participants across racial/ethnic groups as well as subgroups of cognition were compared using independent t-test and Pearson’s chi-square test.

Multiple logistic regressions were performed to define independent risk factors for cognitive impairment and control for potential confounders.

We used STATA 13.0 statistical software for all analysis.

## Results

### Demographic characteristics of study participants

Tables 1 and S3 summarize characteristics of the 541 participants included in this analysis by clinical diagnosis (Table 1), and by race and ethnicity (Table S3). The average age at enrollment was 93.0 ± 2.6 years [range 90–105], and most were female (62.4%). Educational achievement was generally high with most having either some college (40.6%) or graduate school (19.0%) experience. All demographic characteristics differed significantly across the racial/ethnic subgroups. Multiracial and Black individuals had the highest proportion of women (71.7% and 70.4%, respectively) while Asian individuals had the lowest (48.7%). White, Black, and Latino individuals had the same average age (93.2 years) at enrollment, but Asian and Multiracial individuals were significantly younger (92.3 and 92.7 years, respectively). Educational achievement varied significantly by race and ethnicity (Supplement Table 3). The median educational achievement for White, Black and Asian individuals was some level of college education, whereas the median level of educational achievements for Latino individuals was high-school or GED. Health perception and prevalence of vascular risk factors also varied by race/ethnicity with White individuals having the highest degree of perceived health and Black individuals having the greatest prevalence of vascular risk factors.

**Table 1:** Characteristics of participants by cognitive status

Characteristic	NC (*N* = 304)	MCI (*N* = 167)	Dementia (*N* = 70)	*P* value
Demographics				
Age, years, mean (SD)	92.5 (2.3)	93.2 (2.6)	94.4 (3.0)	< 0.001
Age categories				
90–94 years	249 (81.9)	123 (73.7)	35 (50.0)	< 0.001
95–105 years	55 (18.1)	44 (26.3)	35 (50.0)	
Female, *n* (%)	190 (62.5)	103 (61.7)	45 (64.3)	0.931
Education				
≤ HS/GED	106 (35.1)	80 (47.9)	32 (46.4)	0.023
Any college	130 (43.1)	65 (38.9)	22 (31.9)	
Any graduate school	66 (21.9)	22 (13.2)	15 (21.7)	
Health Measures				
Health Perception				
Excellent	23 (7.6)	13 (7.9)	2 (3.0)	0.780^‡^
Very Good	81 (26.9)	38 (23.0)	16 (23.9)	
Good	142 (47.2)	80 (48.5)	34 (50.8)	
Fair	47 (15.6)	28 (17.0)	11 (16.4)	
Poor	8 (2.7)	6 (3.6)	4 (6.0)	
Former Smokers	127 (41.8)	71 (42.5)	24 (34.3)	0.463
Age last smoked, years, mean (SD)	47.3 (16.0)	46.3 (16.1)	48.7 (19.0)	0.842
Medical history				
Cardiovascular disease^c^	140 (47.0)	50 (31.4)	26 (40.0)	0.006
Cerebrovascular disease^d^	44 (14.7)	24 (14.6)	18 (28.1)	0.024
Vascular risk factors^e^	256 (85.9)	120 (75.9)	48 (73.8)	0.008
Physical performance				
SPPB score, mean (SD)	7.0 (3.1)	5.8 (3.2)	4.5 (3.2)	< 0.001
FAQ non-cognitive score, mean (SD)	1.9 (3.2)	3.6 (5.1)	4.8 (4.6)	< 0.001
Cognitive performance				
3MS score, mean (SD)	92 (5.3)	82.8 (7.8)	68.1 (12.4)	< 0.001
FAQ				
Cognitive score, mean (SD)	0.2 (0.5)	1.4 (1.7)	5.9 (4.2)	< 0.001
No. of impaired cognitive items, mean (SD)	0.2 (0–3)	1.3 (1.6)	4.3 (2.6)	< 0.001
CDR global score				
0	256 (84.2)	2 (1.2)	0 (0.0)	< 0.001^‡^
0.5	48 (15.8)	160 (95.8)	16 (22.9)	
1, 2 or 3	0 (0.0)	5 (3.0)	54 (77.1)	
CDR SOB, mean (SD)	0.2 (0.3)	1.5 (1.0)	6.6 (3.5)	< 0.001

*NC* normal cognition, *MCI* mild cognitive impairment, *SPPB* short physical performance battery; *3MS* modified mini-mental state exam; *CDR* clinical dementia rating Scale; *FAQ* functional activities questionnaire

Unless otherwise noted, values are presented as n (%)

Unless otherwise noted, p-values are obtained using chi-square test

^‡^Fisher Exact Test

^c^History of cardiovascular disease includes reported history of any of the following: heart attack, atrial fibrillation, angioplasty/endarterectomy/stent, cardiac bypass surgery, pacemaker/defibrillator, congestive heart failure, angina, and heart valve replacement/repair

^d^History of cerebrovascular disease includes reported history of stroke and/or transient ischemic attack

^e^Vascular risk factors includes reported history of any of the following: hypertension, hypercholesterolemia, and diabetes

### Cognition

Despite exclusion of a prior diagnosis of dementia in the medical record, almost 13% of the individuals evaluated received a clinical diagnosis of dementia at baseline and 31% were diagnosed with MCI.

#### Age, gender, and education in relation to cognition

Initial analysis included demographic characteristics (gender, age, and education). In univariate analyses, increasing age was associated with a higher likelihood of cognitive impairment (*p* < 0.001). College and graduate school achievement were more common among cognitively normal individuals (*p* = 0.023). Gender was not associated with cognitive impairment (*p* = 0.931). In a multiple logistic regression model using these demographic variables, age, college and graduate school education, but not gender, were associated with a higher likelihood of cognitive impairment versus cognitively normal.

#### Race/ethnicity and cognition

There was a significant univariate association between race/ethnicity and cognitive impairment (*p* < 0.02) being highest among Black (57.4%) and lowest among Asian (32.7%) individuals. After adjusting for age, gender, and education, however, there were no race/ethnic differences in the likelihood of cognitive impairment,

#### Vascular disease and cognition

Vascular risk factors (hypertension, hypercholesterolemia, or diabetes) as well as cardiovascular diseases (Table 1) were significantly more prevalent among participants with normal cognition, whereas history of any cerebrovascular disease was significantly more prevalent among the participants with dementia.

### Cognitive performance

#### Association of CDR with diagnosis

As expected, there was a highly significant association between CDR ratings, either as CDR score or CDR sum of boxes (SOB), and clinical diagnosis for this group. Specifically, only 15.8% of individuals determined to be cognitively normal had a CDR score 0.5 as compared to 74.3% of participants with cognitive impairment (Table 1). The association between CDR SOB and diagnosis did not vary after adjusting for age, gender, race/ethnicity, or educational achievement.

#### Cognitive performance measures as predictors of cognitive status

After adjusting for age, gender, education, physical performance (SPPB) and function (non-cognitive FAQ), lower 3MS remained a significant predictor of impaired cognition across all race and ethnic groups. Similarly, higher cognitive FAQ scores remained a significant predictor of cognitive impairment for all subgroups except for Black and multiracial individuals (Table 2).

**Table 2:** Predictors of cognitive impairment by racial/ethnic group*

Covariates	Overall (*n* = 531) OR (95% CI)^a^	White (*n* = 181) OR (95% CI)^a^	Black (*n* = 115) OR (95% CI)^a^	Asian (*n* = 113) OR (95% CI)^a^	Latino (*n* = 76) OR (95% CI)^a^	Multiple (*n* = 46) OR (95% CI)^a^
Demographics						
Age	0.98 (0.87–1.10)	0.95 (0.76–1.18)	0.99 (0.77–1.28)	1.03 (0.79–1.35)	0.80 (0.56–1.16)	0.95 (0.48–1.90)
Gender: women ^b^	1.14 (0.63–2.03)	2.96 (0.98–8.98)	0.26 (0.06–1.12)	1.39 (0.47–4.12)	0.93 (0.16–5.58)	-^d^
Education: any college ^c^	2.61 (0.84–8.07)	1.01 (0.34–3.44)	1.07 (0.25–4.66)	1.31(0.38–4.49)	3.29 (0.41–26.34)	0.65 (0.03–16.28)
Education: any graduate school ^c^	1.95 (0.57–6.7)	0.55 (0.13–2.32)	3.14 (0.26–38.24)	0.57 (0.10–3.32)	0.16, (0.00–274.65)	-^d^
Physical performance						
FAQ non-cognitive score	1.03 (0.95–1.11)	0.98 (0.85–1.14)	1.03 (0.86–1.22)	1.30 (0.98–1.72)	0.95 (0.68–1.31)	0.84 (0.50–1.43)
SPPB score	0.99 (0.90–1.09)	1.01 (0.84–1.22)	0.93 (0.69–1.23)	1.18 (0.96–1.45)	0.98 (0.73–1.32)	0.79 (0.45–1.41)
Cognitive performance						
3MS Score	0.78 (0.74–0.83)	0.75 (0.67–0.85)	0.68 (0.57–0.81)	0.84 (0.76–0.92)	0.83 (0.72–0.95)	0.59 (0.41–0.86)
FAQ cognitive score	3.19 (2.31–4.59)	4.57 (2.29–9.12)	2.05 (0.72–5.86)	2.18 (1.20–3.96)	3.73 (1.56–8.90)	7.58 (0.54–106.37)

*CI* confidence interval, *OR* odds ratio, *SPPB* short physical performance battery; *3MS* modified mini-mental state exam; *FAQ* functional activities questionnaire

*Separate models for each racial/ethnic groups

^a^Derived from logistic regression with cognitive impairment as the outcome and age, gender, education, physical and cognitive performance measures as covariates

^b^Reference group: Men

^c^Reference group: ≤ High school

^d^Not estimable due to small numbers

## Discussion

The *LifeAfter90* Study is an unprecedented epidemiologic study of ethnically diverse oldest-old individuals. The accurate diagnosis of cognitive impairment is critical to assessing the impact of early-life risk and protective factors on dementia incidence and possibly health disparities among diverse populations. Our results indicate that, not only can the diagnosis of cognitive impairment, using accepted clinical diagnostic guidelines [14, 15, 22], be made amongst a racially and ethnically diverse group of individuals 90 years of age and older, but that the utility of “beside” measures of cognitive ability [15], particularly the 3MS and the cognitive component of the FAQ are supported by the results of a detailed and comprehensive clinical evaluation performed in the home. Specifically, lower 3MS remained a significant predictor of impaired cognition across all racial and ethnic groups after adjusting for age, gender, education, and measures of physical performance, suggesting that 3MS and functional disability due to cognitive impairment measured by the FAQ have the potential for use as a screening tool in this population. Moreover, we found that the relationship between scores on these general measures of cognition, function, and clinical diagnosis do not vary by race or ethnicity supporting an unbiased approach to clinical assessment despite widely varying degrees of educational attainment and physical abilities.

### Dementia at baseline

Although individuals were screened by review of their medical histories to exclude prevalent dementia, we expected to enroll some percentage of individuals with dementia. We believe the percentage of individuals with dementia at the baseline evaluation (13%) in this subsample was due partially to lack of regular cognitive surveillance, given the recognized exponential rise in yearly dementia incidence after age 90 [4]. It could also reflect the difficulty of diagnosing dementia among the oldest old in primary care [3].

### Impact of sex differences

Unlike most prevalence studies suggesting higher prevalence in women than in men [5, 25–27], we did not find any significant differences in prevalence of cognitive impairment between men and women in this study. This might be explained by the small sample size and exclusion of the individuals with diagnosis of dementia in their medical record. Further analysis for cognitive impairment among men and women as well as measures of survival after diagnosis of cognitive impairment, will allow for further examination of gender differences in incident dementia among this diverse group of oldest old.

### Vascular risk factor assessment

We found a significantly higher prevalence of vascular risk factors and cardiovascular disease amongst individuals with normal cognition. This result is similar to other studies showing that hypertension and high cholesterol levels have an inverse association with dementia in the oldest-old [28–30]. For this analysis, however, the presence of these diseases was based primarily on participants self-report. Those with normal cognition, therefore, may have been more likely to report these comorbidities compared to those with some cognitive impairment. Additionally, we did not collect information on the duration of, or treatment for, these diseases. Future studies using life-course data obtained from the Kaiser health system will focus on early-life health factors to further understand risk and resilience in this unique cohort. Conversely, a history of cerebrovascular disease (described as transient ischemic attack (TIA) or stroke) was significantly more common among those individuals with dementia consistent with vascular contributions to cognitive impairment as previously noted among participants in the 90 + study[31].

### Strengths and limitations

One of the strengths of this study is the detailed in home assessment performed by the clinicians with clinical adjudication by a behavioral neurologist similar to that used in the East Boston Dementia study [32] and refined by experience in the 90 + study [3]. Importantly, the clinical adjudicator was blind to race and ethnicity. The clinical evaluations were completed in the homes of the participants enabling individuals of all levels of physical and cognitive abilities to participate thereby reducing common forms of selection bias.

The potential limitation of this study might be diagnostic misclassification. This could be due to several reasons, including the difficulty of determining whether functional limitations are due to cognitive impairment or physical limitations (sensory and motor deficits, comorbidities) or the combination of both [3, 12]. Moreover, diagnosis was obtained without assistance of brain imaging or detailed neuropsychological testing, both of which are tools of proven value in the differential diagnosis of dementia [15]. Generalizability of the findings might be another limitation of this study as participants have been long-term members of KPNC system with higher average educational achievement and available access to the health care as compared to the general population.

### Conclusion

In conclusion, the oldest old are a select group of the growing population who may escape major illnesses or delay onset toward the end of life. Preliminary analysis of this population finds excellent consistency between “bedside”[15] cognitive and functional measures and the clinical diagnosis of cognitive impairment blindly adjudicated by a behavioral neurologist that were unaffected by the race or ethnicity of the participants. Lifestyle, health, and genetic factors that could be of great importance to understanding dementia disparities in this oldest-old population will be further explored once enrollment is complete and the clinical diagnostic data is linked with the decades of prospectively collected health information and detailed neuropsychological testing available for the participants of *LifeAfter90* study.

## Supplementary Information

Below is the link to the electronic supplementary material.

Supplementary file1 (DOCX 38 KB)

## Data Availability

The funder had no role in study design, subject recruitment, data collection and analysis, and preparation of the manuscript.

## Declarations

### Human and animal participants

All procedures performed in this study involving human participants were in accordance with the ethical standards of the institutional and/or national research committee and with the 1964 Helsinki Declaration and its later amendments or comparable ethical standards. He study was approved by the Bioethics Committee of the University of California at Davis on 2/18/2018 under IRB approved protocol #1180809. All subjects signed informed consent prior to enrollment in this study.
