# Supplementary material for: Assessing cognitive impairment in an ethnically diverse cohort of oldest-old: the life after 90 study
Source: Aging Clin Exp Res. 2023 Mar 4;35(5):979–86. doi: 10.1007/s40520-023-02368-0 (PMC10149459; doi:10.1007/s40520-023-02368-0)
Supplement: Supplementary file 1 — Supplementary file1 (DOCX 38 KB) [file 40520_2023_2368_MOESM1_ESM.docx]

**SUPPLEMENTARY TABLE S1: UC Davis Data Collection Instruments**

|  | **Description** | **Scoring** | **Source** | | **Frequency** | |
| --- | --- | --- | --- | --- | --- | --- |
|  |  |  | **Participant** | **Informant** | **Repeated**  **(2 x per year)** | **single** |
| Health history, physical & neurological examination | Completed by physicians trained in cognitive impairment assessment | NA | X |  | X |  |
| Clinical Dementia Rating Scale (CDR) | Washington University Clinical Dementia Rating based on the semi-structured interview and assesses each of the 6 cognitive categories (memory, orientation, judgement and problem solving, community affairs, home and hobbies, and personal care) | 5-point scale where:  0 – no impairment  0.5 – questionable impairment  1 – mild impairment  2 – moderate impairment  3 – severe impairment | X | X | X |  |
| Modified Mini-Mental State (3MS) Exam | Offers a brief assessment of attention, concentration, orientation to time and place, short- and long-term memory, language ability, constructional praxis, abstract thinking and list-generating frequency | 100-point scale with higher scores indicating better performance | X |  | X |  |
| Functional Activities Questionnaire (FAQ) | Collects information IADLs (12 domains). Modified FAQ allows distinction between functional impairment due to cognition and other physical reasons such as hearing, vision, issues in UE, LE and balance. | Total score: 36-point score  Cognitive score: 24-point scale  Non-Cognitive score: 60-point scale  The higher FAQ scores indicate more functional limitations | X | X | X |  |
| Short Physical Performance Battery (SPPB) | Objective, age appropriate assessment of the gait and balance | 12-point scale with higher scores indicating better physical performance | X |  | X |  |
| Blood and/or Saliva sample | APoE genotyping | NA | X |  |  | X |
| MRI and/or PET scan | To characterize cerebral amyloid burden, vascular lesions, and atrophy | NA | X |  |  | X |
| Dementia Questionnaire (DQ) | Assesses the presence of cognitive and functional impairment, it’s onset and course | NA |  | X | X* | X** |

Abbreviations: IADL=instrumental activities of daily living, UE=upper extremities, LE=lower extremities

^*^ Completed when the participant cannot complete the assessment (too impaired physically and/or cognitively)

^**^Post-mortem DQ is completed only once

**SUPPLEMENTARY TABLE S2: Modified Functional Assessment Questionnaire**

|  | Ability | If ability not normal, code: | | | | | |
| --- | --- | --- | --- | --- | --- | --- | --- |
|  |  | Cognition | Hearing Loss | Vision Loss | Upper Extremities | Lower Extremities | Other Physical |
| Functional Abilities | 0=Normal/Does not do, but could  1=Difficulty/Did, but could not  2=Needs Assistance  3=Dependent | 0=No  1=Difficulty  2=Loss | 0=No  1=Yes | 0=No  1=Yes | 0=No  1=Yes | 0=No  1=Yes | 0=No  1=Yes |
| 1. Writing checks, paying bills, balancing a checkbook |  |  |  |  |  |  |  |
| 1. Shopping alone for clothes, groceries, household necessities |  |  |  |  |  |  |  |
| 1. Keeping track of current events |  |  |  |  |  |  |  |
| 1. Travelling out of the neighborhood, arranging to take a bus or taxi |  |  |  |  |  |  |  |
| 1. Feeding, including cutting meat or buttering bread |  |  |  |  |  |  |  |
| 1. Bathing, either sponge bath, tub bath, shower |  |  |  |  |  |  |  |
| 1. Dressing, including getting clothes from closets and drawers and using fasteners |  |  |  |  |  |  |  |
| 1. Getting in and out of the bed or chair |  |  |  |  |  |  |  |
| 1. Going to the toilet and cleaning self after |  |  |  |  |  |  |  |
| 1. Controlling urination and bowel movements |  |  |  |  |  |  |  |
| 1. Moving indoors |  |  |  |  |  |  |  |
| 1. Taking medications |  |  |  |  |  |  |  |
| Comments |  | | | | | | |
| Source of information | - Participant | | - Informant | | - Examiner’s judgment | | |

**SUPPLEMENTARY TABLE S3: Characteristics of Participants by Race/Ethnicity**

| Characteristic | Total No.^a^ | All participants  (N=535) | White  (n=183) | Black  (n=115) | Asian  (n=113) | Latino  (n=78) | Multiracial  (n=46) | P-value |
| --- | --- | --- | --- | --- | --- | --- | --- | --- |
| Demographics |  |  |  |  |  |  |  |  |
| *Age, yr, mean (SD)* | 535 | 93.0 (2.6) | 93.2 (2.6) | 93.2 (2.8) | 92.3 (2.1) | 93.2 (2.8) | 92.7 (2.3) | 0.029 |
| *Women* | 535 | 334 (62.4) | 114 (62.3) | 81 (70.4) | 55 (48.7) | 51 (65.4) | 33 (71.7) | 0.007 |
| *Education* | 532 |  |  |  |  |  |  | <0.001 |
| ≤HS/GED |  | 215 (40.4) | 54 (29.8) | 56 (48.7) | 34 (30.1) | 51 (66.2) | 20 (43.5) |  |
| Any college |  | 216 (40.6) | 77 (42.5) | 47 (40.9) | 52 (46.0) | 21 (27.3) | 19 (41.3) |  |
| Any graduate school |  | 101 (19.0) | 50 (27.6) | 12 (10.4) | 27 (23.9) | 5 (6.5) | 7 (15.2) |  |
| Health Measures |  |  |  |  |  |  |  |  |
| *Health Perception* | 527 |  |  |  |  |  |  | <0.001^b^ |
| Excellent |  | 37 (7.0) | 22 (12.1) | 1 (1.8) | 4 (3.6) | 8 (10.5) | 1 (2.2) |  |
| Very good |  | 134 (25.4) | 58 (31.9) | 27 (23.9) | 21 (19.1) | 18 (23.7) | 10 (21.7) |  |
| Good |  | 253 (48.0) | 73 (40.1) | 57 (50.4) | 63 (57.3) | 37 (48.7) | 23 (50.0) |  |
| Fair |  | 85 (16.1) | 25 (13.7) | 21 (18.6) | 17 (15.5) | 11 (14.5) | 11 (23.9) |  |
| Poor |  | 18 (3.4) | 4 (2.2) | 6 (5.3) | 5 (4.5) | 2 (2.6) | 1 (2.2) |  |
| *Former smokers* | 532 | 217 (40.8) | 85 (46.4) | 53 (46.9) | 34 (30.4) | 29 (37.2) | 16 (34.8) | 0.035 |
| *Age, last smoked, yr, mean (SD)* | 217 | 47.2 (16.3) | 45.8 (15.6) | 45.9 (16.4) | 49.5 (18.5) | 47.0 (15.9) | 53.1 (15.4) | 0.457 |
| *Medical History* |  |  |  |  |  |  |  |  |
| Cardiovascular disease^c^ | 516 | 214 (41.5) | 76 (42.7) | 39 (36.1) | 50 (45.0) | 26 (34.7) | 23 (52.3) | 0.238 |
| Cerebrovascular disease^d^ | 522 | 84 (16.1) | 35 (19.7) | 17 (15.5) | 10 (8.9) | 14 (18.2) | 8 (17.8) | 0.177 |
| Vascular risk factors^e^ | 515 | 418 (81.2) | 123 (71.1) | 102 (92.7) | 95 (85.6) | 59 (76.6) | 39 (88.6) | <0.001 |
| Clinical Diagnosis | 535 |  |  |  |  |  |  | 0.009 |
| NC |  | 301 (56.3) | 109 (59.6) | 49 (42.6) | 76 (67.3) | 42 (53.9) | 25 (54.4) |  |
| MCI |  | 165 (30.8) | 55 (30.1) | 47 (40.9) | 27 (23.9) | 20 (25.6) | 16 (34.8) |  |
| Dementia |  | 69 (12.9) | 19 (10.4) | 19 (16.5) | 10 (8.9) | 16 (20.5) | 5 (10.9) |  |
| Physical Performance |  |  |  |  |  |  |  |  |
| *SPPB score, mean (SD)* | 535 | 6.3 (3.2) | 6.5 (3.1) | 5.5 (3.1) | 7.4 (3.2) | 6.1 (3.3) | 4.9 (3.1) | <0.001 |
| *FAQ non-cognitive score, mean (SD)* | 534 | 2.8 (4.2) | 2.8 (3.9) | 3.3 (5.3) | 1.8 (3.4) | 3.0 (3.3) | 3.8 (5.0) | <0.001 |
| *Cognitive Performance* |  |  |  |  |  |  |  |  |
| *3MS score, mean (SD)* | 535 | 86.1 (11.0) | 88.6 (9.6) | 82.9 (12.6) | 87.9 (8.2) | 81.6 (13.4) | 87.5 (8.8) | <0.001 |
| *FAQ* |  |  |  |  |  |  |  |  |
| Cognitive score, mean (SD) | 534 | 1.3 (2.6) | 1.1 (2.1) | 1.6 (3.1) | 1.1 (2.4) | 2.2 (3.6) | 0.8 (1.4) | 0.005 |
| No. of impaired cognitive items, mean (SD) | 534 | 1.1 (1.9) | 0.9 (1.6) | 1.3 (2.2) | 0.9 (1.9) | 1.7 (2.3) | 0.7 (1.2) | 0.010 |
| *CDR global score* | 535 |  |  |  |  |  |  | 0.001 |
| 0 |  | 255 (47.7) | 93 (50.8) | 42 (36.5) | 69 (61.1) | 31 (39.7) | 20 (43.5) |  |
| 0.5 |  | 221 (41.3) | 74 (40.4) | 58 (50.4) | 36 (31.9) | 30 (38.5) | 23 (50.0) |  |
| 1, 2 or 3 |  | 59 (11.0) | 16 (8.7) | 15 (13.0) | 8 (7.1) | 17 (21.8) | 3 (6.5) |  |
| *CDR SOB, mean (SD)* | 535 | 1.4 (2.5) | 1.3 (2.4) | 1.7 (2.6) | 1.0 (2.0) | 2.3 (3.6) | 1.0 (1.3) | <0.001 |
| Unless otherwise noted, values are presented as n (%)  NC=Normal Cognition, MCI=Mild Cognitive Impairment, SPPB=Short Physical Performance Battery; 3MS=Modified Mini-Mental State Exam; CDR=Clinical Dementia Rating Scale; FAQ=Functional Activities Questionnaire  ^a^ The total number is less than 541 due to the undisclosed information on race/ethnicity for 6 participants  ^b^ Fisher Exact Test  ^c^ History of cardiovascular disease includes reported history of any of the following: heart attack, atrial fibrillation, angioplasty/endarterectomy/stent, cardiac bypass surgery, pacemaker/defibrillator, congestive heart failure, angina, and heart valve replacement/repair  ^d^ History of cerebrovascular disease includes reported history of stroke and/or transient ischemic attack  ^e^ Vascular risk factors includes reported history of any of the following: hypertension, hypercholesterolemia, and diabetes | | | | | | | | |
